# Weight loss, diet composition and breast cancer incidence and outcome in postmenopausal women

**DOI:** 10.18632/oncotarget.26864

**Published:** 2019-05-03

**Authors:** Kathy Pan, Juhua Luo, Aaron K. Aragaki, Rowan T. Chlebowski

**Affiliations:** ^1^ Department of Medical Oncology and Hematology, Los Angeles Biomedical Research Institute, Torrance, CA, USA; ^2^ Department of Epidemiology and Biostatistics, Indiana University Bloomington, Bloomington, IN, USA; ^3^ Department of Public Health Sciences, Fred Hutchinson Cancer Research Center, Seattle, WA, USA

**Keywords:** breast cancer, obesity, weight loss, waist circumference, Women's Health Initiative

## Abstract

Two complementary studies in separate components of the Women’s Health Initiative (WHI) examined relationships among weight loss, diet composition and breast cancer incidence and outcome in postmenopausal women. In the WHI Observational Study, 61,335 postmenopaus al women had their weight change determined over a 3-year period with subsequent follow-up. Women with weight loss greater than or equal to 5% had significantly lower breast cancer incidence compared to women with stable weight. In the WHI Dietary Modification randomized clinical trial involving 48,835 postmenopausal women, implementation of a low-fat eating pattern significantly reduced deaths after breast cancer. Thus, moderation regarding dietary composition and body weight maintenance can reduce a postmenopausal woman’s risk of being diagnosed with breast cancer and of dying after breast cancer.

## BACKGROUND

### The Women’s Health Initiative Observational Study (WHI OS)

Obesity has become a major health problem for the 40% of postmenopausal women in the US who are currently obese (body mass index [BMI] ≥ 30 kg/m2) [1]. The International Agency for Research on Cancer (IARC), based on strong and consistent observational study evidence, has linked 13 cancer sites to obesity [2]. Breast cancer, one of the cancers strongly associated with obesity, is the most common cancer in postmenopausal women. However, the clinically relevant question of whether breast cancer risk can be reduced by weight loss in adult life has remained unanswered as observational studies have provided inconsistent results [3]. As it has been established that weight loss of 5% can be achieved and maintained for several years [4], we examined the influence of a similar magnitude of weight loss on breast cancer incidence in postmenopausal women participating in the Women’s Health Initiative (WHI) Observational Study (OS) [5].

The study population included 61,335 postmenopausal women, entered between 1993 and 1998 at 40 US clinical centers, who were aged 50–79 years and had no prior breast cancer with a normal baseline mammogram. Anthropometrics were measured and BMI calculated at baseline and year 3. Weight change at year 3 was categorized as stable (<5% change), loss (≥5% decrease) or gain (≥5% increase). Participants were followed annually for clinical outcomes. Breast cancer incidence was determined through 11.4 years (mean) follow-up after the year 3 weight status assessment and all breast cancers were verified by central medical record review. Survival findings were augmented by serial National Death Index (NDI) queries, which capture 98% of all deaths [6].

Among 61,335 participants, 3,061 developed breast cancer. Women with weight loss (≥ 5%) had a significantly lower breast cancer incidence compared to women with stable weight (hazard ratio [HR] 0.88, 95% confidence interval [CI] 0.78–0.98, P = 0.02), and the findings did not differ by whether the weight loss was intentional or not. We examined associations between weight change category and breast cancer risk by stratifying variables: baseline BMI, race/ethnicity, age, and menopausal hormone therapy, with none of the interactions found to be statistically significant. Weight gain was not associated with higher breast cancer risk overall, although a higher risk of triple-negative breast cancer was seen (HR 1.54, 95% CI 1.16–2.05).

In addition and not previously reported, we examined the change in waist circumference (WC) at year 3 from baseline with subsequent breast cancer risk. The study population was 60,552 after excluding those with missing WC, and 3,379 breast cancer cases were identified.

The association between change in WC and subsequent breast cancer risk was examined using the Cox proportional hazards regression model, and the proportional hazards assumption was checked and held. The HRs and 95% CIs for the association between WC category (stable WC [within 5%], WC gain [≥5%], WC loss [≥5%]) and breast cancer risk are shown in Table 1. No statistically significant associations were detected between WC change and breast cancer incidence. When examined by baseline BMI and tumor subtype, a lower risk of breast cancer in women with WC loss was seen only for overweight women and for progesterone receptor-positive breast cancer (data not shown).

**Table 1:** Hazard ratios (HR) and 95% CIs for the association between WC change category and risk of breast cancer

	Cases	Age-adjusted HR (95% CI)	Multivariable-adjusted^a^ HR (95% CI)
**WC change between baseline and year 3 (percentage change)**			
Stable WC (within ±5% change)	2004	Reference	Reference
WC gain (≥5%)	875	0.98 (0.91 1.06)	1.00 (0.92 1.08)
WC loss (≥5%)	500	0.98 (0.89 1.08)	0.94 (0.85 1.04)
Intentional	346	0.96 (0.86 1.08)	0.92 (0.82 1.03)
Unintentional	154	1.02 (0.87 1.20)	1.00 (0.85 1.18)

a. In multivariable models, we adjusted for Gail score (based on age, race/ethnicity, age at menarche, age of the mother at the birth of her first live child, number of first-degree relatives with breast cancer, and the number of previous breast biopsy examinations) [8], education, smoking pack-years, recreational physical activity, alcohol, history of hormone therapy use, parity, BMI, and waist circumference.

A cross tabulation of changes in WC and changes in weight is listed in Table 2. Changes in WC did not correlate with changes in weight. For example, among the women who had ≥5% intentional WC loss, the majority (59.25%) had stable weight, whereas 35.85% had intentional ≥5% weight loss. On the other hand, among the women who had ≥5% intentional weight loss, approximately equal proportions had stable WC and intentional ≥5% WC loss (45.94% and 47.82%, respectively). Although measurements were obtained in standard fashion by trained personnel in the WHI, measurement error in WC is a known limitation of studies incorporating this measure [7].

**Table 2:** Waist circumference (WC) change category by weight change category

WC change	Weight change				
Frequency Percent Row Pct Col Pct	Stable weight (±5%)	Weight gain (≥5%)	Weight loss (≥5%) - Unintentional	Weight loss (≥5%) -Intentional	Total
**Stable waist circumference (WC) (**±**5%)**	26948	4654	1516	2193	35311
	44.50	7.69	2.50	3.62	58.32
	76.32	13.18	4.29	6.21	
	66.31	39.32	45.86	45.94	
**WC gain (**≥**5%)**	8604	6774	148	298	15824
	14.21	11.19	0.24	0.49	26.13
	54.37	42.81	0.94	1.88	
	21.17	57.24	4.48	6.24	
**WC loss (**≥**5%) - Unintentional**	1312	94	1642	0	3048
	2.17	0.16	2.71	0.00	5.03
	43.04	3.08	53.87	0.00	
	3.23	0.79	49.67	0.00	
**WC loss (**≥**5%) - Intentional**	3773	313	0	2283	6369
	6.23	0.52	0.00	3.77	10.52
	59.24	4.91	0.00	35.85	
	9.28	2.64	0.00	47.82	
**Total**	40637	11835	3306	4774	60552
	67.11	19.55	5.46	7.88	100.00

The finding that weight loss determined over a relatively short 3-year period was associated with a significantly lower breast cancer risk differs from results from other observational studies, which are commonly based on weight determinations over longer periods. We speculate that, as older women (>70 years of age) tend to lose weight, longer periods of weight measure may dilute a potential breast cancer benefit of short-term weight loss at an earlier age. In any event, while the substantial weight loss seen following bariatric surgery has been associated with lower breast cancer risk [9], the WHI analysis findings indicate that even smaller degrees of weight loss may reduce breast cancer risk and support a public health message of potential cancer reduction benefit of weight loss in postmenopausal women.

## ASSOCIATED CLINICAL STUDIES

### WHI OS and endometrial cancer risk

Weight loss has also been associated with significantly lower risk endometrial cancer risk when similarly evaluated in the WHI OS. In this analysis, women with hysterectomy before enrollment (n = 33,317) and women with prior endometrial cancer were excluded, leaving 36,793 women in the final analysis. Women with weight loss ≥5% had significantly lower endometrial cancer risk compared to women with stable weight (HR 0.71, 95% CI 0.54–0.95), with the association strongest among obese women with intentional, rather than unintentional, weight loss (HR 0.44, 95% CI 0.25–0.78) [10], a finding not seen in analyses examining breast cancer associations with weight loss [11]. Further studies are needed to determine the differential influence of intentional versus unintentional weight loss on cancer outcomes in postmenopausal women.

### Women’s Health Initiative Dietary Modification randomized trial

In the WHI Dietary Modification (DM) trial, 48,835 postmenopausal women, all with no prior breast cancer and normal baseline mammogram, were randomized to a dietary intervention (a low-fat eating pattern incorporating an increase in fruit, vegetables and grain intake) or to a usual diet comparison group between 1993 and 1998. Breast cancer and colorectal cancer were separate primary study endpoints [11]. The dietary modification group participated in 18 group sessions led by centrally trained nutritionists in year 1 and quarterly sessions throughout the dietary intervention period. Caloric restriction or weight loss were not intervention targets. After 1 year, all dietary targets were significantly and favorably changed in the dietary group, and there was a statistically significant 3% weight loss that remained significant at years 3 and 6 (all P < 0.001) but attenuated over time [11, 12].

After 8.5 years (median) dietary intervention, among the 1764 incident breast cancer cases, the incidence rate was 8% lower in the dietary intervention group, a finding which was not statistically significant (P = 0.09) [11]. However, during that dietary intervention period, a statistically significant reduction in deaths after breast cancer (breast cancer followed by death from any cause) was seen (P = 0.01) [13]. Specifically, after 16.1 years cumulative follow-up, with 3,030 incident breast cancers, deaths after breast cancer continued to be significantly reduced (HR 0.82, 95% CI 0.70–0.96) in the dietary group [13]. In further analysis of this trial, breast cancer overall survival (breast cancer diagnosis followed by death from any cause, measured from the time of breast cancer diagnosis) was found to be significantly increased for intervention versus comparison group participants for breast cancers diagnosed during the dietary period (10-year survival of 82% versus 78% for intervention versus comparison, respectively, P = 0.01) [14]. The dietary intervention had a greater benefit among obese participants for deaths after breast cancer [13] and breast cancer overall survival [14].

While the dietary intervention goal was to reduce fat intake to 20% of total energy, at one year, mean energy from fat was actually reduced to 24.3% (standard deviation [SD] 7.5%) [11] and was 29.8% at the end of the dietary intervention [12]. The magnitude of this intervention has been previously described as representing “a modest reduction in fat intake with minimal weight loss” which “could be easily achievable by many” [13].

The important question of whether dietary change or weight loss will alter outcome for women following a diagnosis of early-stage breast cancer [15] was not directly addressed in the WHI DM trial. As all participants were free of cancer at entry, depending on their subsequent date of diagnosis, women could have either more time on the dietary intervention before or following diagnosis. As it appeared that exposure to the dietary intervention after breast cancer diagnosis had greater effect on breast cancer mortality [14], the study findings could have relevance to the adjuvant breast cancer setting as well.

The association between implementation of a low-fat eating pattern and reduction in deaths after breast cancer was unchanged by the addition of baseline weight and weight change to the analysis [13, 14]. As weight loss was not an intervention target in the trial, the WHI DM trial did not evaluate the hypothesis raised by the WHI Observational Study finding that associated ≥5% weight loss with lower breast cancer incidence [5]. However, the modest 3% weight loss seen in WHI DM dietary participants suggests nutritional composition change rather than weight loss was the driver of the favorable breast cancer outcome. Ongoing studies are exploring this question.

## SUMMARY

In summary, complementary findings from the WHI OS and the WHI DM full-scale randomized cancer prevention clinical trials indicate two distinct routes to improve breast cancer outcomes. A low-fat diet rich in vegetables and fruits can improve breast cancer outcome. In addition, for overweight or obese postmenopausal women, modest weight loss can improve breast cancer outcomes, and may enhance the benefits of a healthy low-fat diet. The current message should be encouraging for postmenopausal women, namely, moderation regarding dietary composition and weight can significantly reduce a woman’s chance of being diagnosed with breast cancer and of dying after a breast cancer diagnosis.

## WOMEN’S HEALTH INITIATIVE INVESTIGATORS

### Program office

Jacques Roscoe, Shari Ludlum, Dale Burden, Joan McGowan, Leslie Ford, and Nancy Geller - National Heart, Lung, and Blood Institute, Bethesda, MD, USA.

### Clinical coordinating center

Garnet Anderson, Ross Prentice, Andrea LaCroix, and Charles Kooperberg - Fred Hutchinson Cancer Research Center, Seattle, WA, USA.

### Investigators and academic centers

JoAnn E. Manson - Brigham and Women’s Hospital, Harvard Medical School, Boston, MA, USA; Barbara V. Howard - MedStar Health Research Institute/Howard University, Washington, DC, USA; Marcia L. Stefanick - Stanford Prevention Research Center, Stanford, CA, USA; Rebecca Jackson - The Ohio State University, Columbus, OH, USA; Cynthia A. Thompson - University of Arizona, Tucson/Phoenix, AZ, USA; Jean Wactawski-Wende - University at Buffalo, Buffalo, NY, USA; Marian Limacher - University of Florida, Gainesville/Jacksonville, FL, USA; Robert Wallace - University of Iowa, Iowa City/Davenport, IA, USA; Lewis Kuller - University of Pittsburgh, Pittsburgh, PA, USA; Rowan T. Chlebowski - Los Angeles Biomedical Research Institute, Torrance, CA, USA; Sally Shumaker - Wake Forest University School of Medicine, Winston-Salem, NC, USA.
